# Uncoiling Mechanics of Escherichia coli Type I Fimbriae Are Optimized for Catch Bonds

**DOI:** 10.1371/journal.pbio.0040298

**Published:** 2006-08-29

**Authors:** Manu Forero, Olga Yakovenko, Evgeni V Sokurenko, Wendy E Thomas, Viola Vogel

**Affiliations:** 1 Department of Materials, Laboratory for Biologically Oriented Materials, ETH Zurich, Zurich, Switzerland; 2 Department of Bioengineering, University of Washington, Seattle, Washington, United States of America; 3 Department of Microbiology, University of Washington, Seattle, Washington, United States of America; Stanford University, United States of America

## Abstract

We determined whether the molecular structures through which force is applied to receptor–ligand pairs are tuned to optimize cell adhesion under flow. The adhesive tethers of our model system, *Escherichia coli,* are type I fimbriae, which are anchored to the outer membrane of most *E. coli* strains. They consist of a fimbrial rod (0.3–1.5 μm in length) built from a helically coiled structural subunit, FimA, and an adhesive subunit, FimH, incorporated at the fimbrial tip. Previously reported data suggest that FimH binds to mannosylated ligands on the surfaces of host cells via catch bonds that are enhanced by the shear-originated tensile force. To understand whether the mechanical properties of the fimbrial rod regulate the stability of the FimH–mannose bond, we pulled the fimbriae via a mannosylated tip of an atomic force microscope. Individual fimbriae rapidly elongate for up to 10 μm at forces above 60 pN and rapidly contract again at forces below 25 pN. At intermediate forces, fimbriae change length more slowly, and discrete 5.0 ± 0.3–nm changes in length can be observed, consistent with uncoiling and coiling of the helical quaternary structure of one FimA subunit at a time. The force range at which fimbriae are relatively stable in length is the same as the optimal force range at which FimH–mannose bonds are longest lived. Higher or lower forces, which cause shorter bond lifetimes, cause rapid length changes in the fimbria that help maintain force at the optimal range for sustaining the FimH–mannose interaction. The modulation of force and the rate at which it is transmitted from the bacterial cell to the adhesive catch bond present a novel physiological role for the fimbrial rod in bacterial host cell adhesion. This suggests that the mechanical properties of the fimbrial shaft have codeveloped to optimize the stability of the terminal adhesive under flow.

## Abstract 2

Bacterial infection relies on successful adhesion of the fimbrial rod to the host cell under all physiological conditions. Atomic force microscopy reveals how the rod uncoils in response to variable forces.

## Introduction

A critical step in bacterial infection is the attachment of the bacterium to a host surface, which is often mediated by specific interactions between adhesive proteins called adhesins and their target receptors on host tissues. This attachment usually occurs in the presence of flowing bodily fluids, which create drag forces on bacteria and their anchoring adhesins. FimH is the most common adhesin for Escherichia coli and other enteric bacteria, and it binds specifically to the carbohydrate mannose. Although it might be expected that high flow would wash off bound bacteria, we have previously shown that forces generated by fluid drag on bacteria enhance FimH-mediated bacterial attachment to cells and surfaces [1–3]. Our previous work supports the notion that the main cause of this enhanced attachment is a force-enhanced conformational switch from a short-lived (weak) to a long-lived (strong) state of the FimH–mannose complex [4]. At high shear, even the long-lived FimH–mannose bonds break [3,4]. Thus, force has a biphasic effect on FimH–mannose bond strength, where maximal adhesion is seen at a specific level of force [1–4], and the bond lifetime drastically drops at higher and lower forces [4]. Because the level of force applied to FimH–mannose bonds is critical to the adhesion behavior of *E. coli,* it is important to understand how the force acting on the bacterium is transmitted to the bonds.

FimH, like many adhesins in Gram-negative bacteria, is not directly attached to the bacterial wall, but is exposed at the tips of long filamentous organelles, named fimbriae or pili, that are anchored to the outer membrane. Proposed functions of the fimbrial rod include extending the reach of adhesins beyond the bacterial capsule [5] and overcoming repulsion from surfaces [6], but the physiological significance of the adhesin-presenting fimbrial rod remains unclear. The length of fimbriae is much larger than the range of electrostatic repulsion, because attractive Van der Waals forces dominate beyond 50 nm [7]. Type I fimbriae are 7 nm in diameter and are ~1-μm-long polymer rods composed of structural protein subunits called FimA, which weigh 15.7 kDa [8,9]. By homology with FimH, FimA is expected to be a β barrel (immunoglobulin [Ig]-like fold) [8,9]. FimA subunits are interlinked via β strand swapping: a protruding β strand from one subunit replaces a missing β strand of the β barrel of the following subunit, stabilizing its tertiary structure [9–11]. This linear polymer chain forms a right-handed helical rod, where each turn is made of 3.4 FimA subunits and has a pitch of 2.4 nm [9]. None of the previously proposed functions of fimbriae addresses why they are made from a coiled polymer. The quaternary structure of type I fimbriae has been suggested to uncoil in glycerol [12], but it is unclear if this happens under physiological conditions. Recent experiments with optical tweezers suggested that P-pili, which are thinner and more flexible than type I fimbriae but apparently have a similar helical structure, could uncoil and coil reversibly [13,14]. However, the dynamics of the process were not studied and no specific ligands were used to induce uncoiling. Thus, it has not been possible to predict how the mechanical properties of the fimbrial shaft will modulate the amount of force applied to the terminal adhesin and affect the function of the adhesin–ligand bonds at the fimbrial tips.

We used an atomic force microscope (AFM) to pull on type I fimbriae to show elongation and contraction at a wide range of physiological forces. We attribute these changes in length to sequential uncoiling of the quaternary structure of fimbriae, and we confirmed this by showing coiling and uncoiling events of individual FimA subunits at intermediate forces. Furthermore, by probing the lifetimes of the terminal adhesin at intermediate to high forces with the AFM and in flow chamber experiments, we conclude that significant length changes due to coiling or uncoiling happen at forces where the lifetime of the terminal catch bond is reduced (low and high forces), whereas no significant changes in length are observed for forces at which bonds are strongest (intermediate forces). We propose that the mechanical properties of the terminal adhesin and fimbrial proteins have codeveloped to confer adhesive advantages to bacteria.

## Results

### Fimbriae Extend under Force Applied via the Terminal Adhesin-Bound Ligand

**Figure 1:**
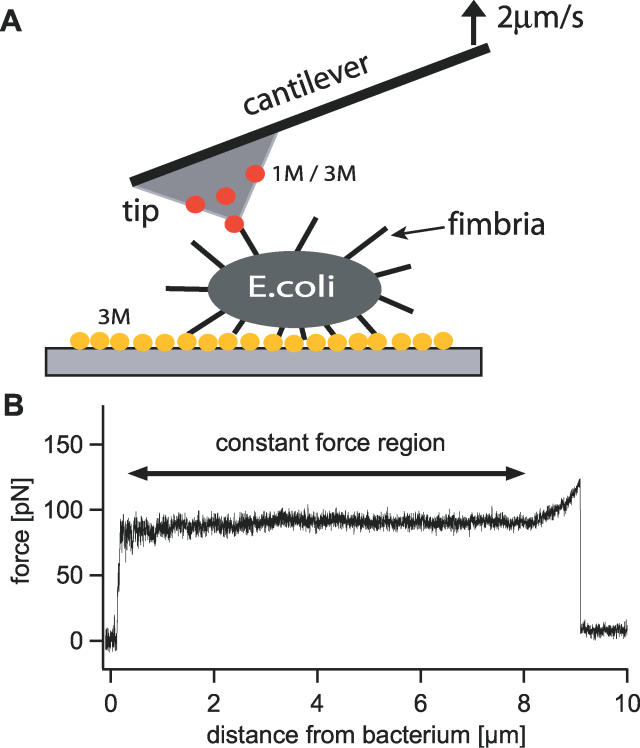
Force Measurements on Type I Fimbriae of E. coli (A) Experimental setup. The tip (gray) of an AFM cantilever is coated with 1M-BSA (red) or 3M-RNAseB (yellow). An E. coli bacterium is attached to a 3M-covered glass surface. After the bacterium is approached with the AFM tip, one or multiple fimbriae can bind. The cantilever base is then pulled away from the bacterium at a constant velocity. (B) Force/extension curve for a single fimbria measured at a constant velocity of 2 μm/s. When pulled at various constant velocities, fimbriae extend to multiple times their original length of ~0.7 μm. The force profile shows three distinct regions: first, the force increases quickly with separation. Second, this is followed by a constant-force region where the AFM tip can be pulled away for several micrometers. Third, the force then increases nonlinearly before the bond between the mannose coated AFM tip and the fimbria finally breaks. (The spring constant of the cantilever used in this pull was 7.9 pN/nm.)

**Figure 2:**
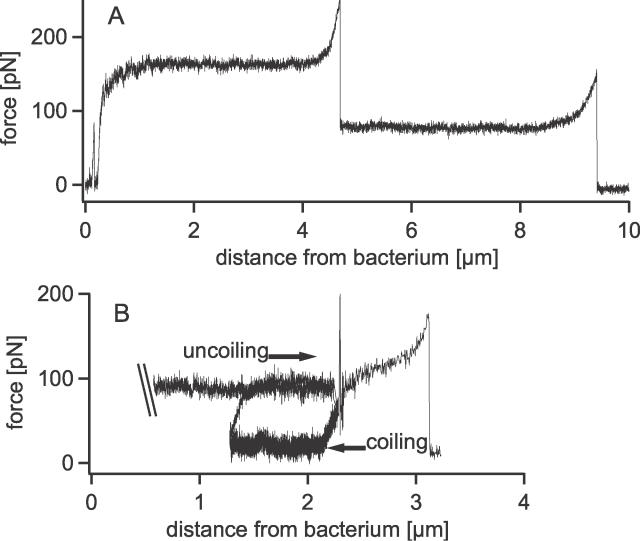
Force/Extension Curves of Fimbriae (A) Force/extension curve when two fimbriae attach to a mannose-coated tip retracting at 2 μm/s. After the first fimbria snaps off the tip, the second fimbria continues to be attached and is pulled until it detaches. The pulling force in the first constant-force region is close to twice that in the second constant-force region, and two distinct break events are observed, indicating that individual fimbriae are being probed. (B) Reversible uncoiling and coiling of a single fimbria. After pulling a fimbria for a set distance, the pulling direction was reversed and the fimbria was observed to coil back at a nonzero force. After a second reversal to the initial pulling velocity, the force returned to its original level. These data show that both uncoiling and coiling are sequential and reversible. The spring constant of the cantilever used in these pulls was 7.9 pN/nm, and the pulls were done on two different bacteria. Most of the >5,000 successful constant velocity pulls we observed are qualitatively similar to those in Figures 1B or 2A with two (shown here) or more fimbriae. The main difference was in the length of the constant-force region as expected from the variability of the length of fimbriae.

The cantilever of an AFM functionalized with monomannose (1M) or trimannose (3M) was brought into contact with surface-immobilized bacteria by applying a pushing force of 50–200 pN. Subsequently the cantilever base was retracted at a constant velocity of 2 μm/s by using the closed-loop mode of the AFM to ensure a constant velocity (Figure 1A). During retraction, the force on the cantilever tip was measured to obtain a force/extension curve (Figures 1B and 2) of type I pili. These force/extension curves present three different force regions before breaking. Closer to the bacterium, the force increases with the separation between the AFM tip and the bacterium. This is followed by a region of a few micrometers in length for which the force remains essentially constant with increasing separation (constant-force region) (Figure 1B). Finally, there is a section in which force increases nonlinearly with separation until the interaction between the AFM tip and the bacterium breaks. Addition of 4% α-methylmannoside to the buffer reduced by 85% the number of all types of adhesive events observed between the functionalized AFM tip and fimbriae. Thus, the connection between the mannosylated AFM cantilever and bacteria is specific and occurs via the fimbrial tip–associated FimH proteins.

### Rupture Reflects Breakage of Single FimH–Mannose Bonds

To check that the long constant-force region is not due to drag caused by bacterial detachment from the surface or uncoiling from pili on the surface side of bacteria, multiple bacteria were followed by mounting the AFM over an optical microscope with a 100× phase-contrast oil-immersion objective. After more than 200 pulls on the same bacterium, the bacterium did not separate from the surface, even when force events spanning several micrometers were recorded by the AFM. Furthermore, during multiple mannose-specific AFM pulls of the same cell, breaks happened repeatedly at particular distances away from the bacterium. This result suggests that the same fimbriae were pulled repeatedly and strongly, suggesting that the breaks are due to the rupture of the specific FimH–mannose interaction rather than to a breakage of the fimbriae, because a broken fimbria would not be available to rebind. Sometimes, multiple fimbriae were clearly attached to the AFM tip, as reflected in both the number of independent breaking events and the total uncoiling force being proportional to the number of attached fimbriae (Figure 2A). Because the breaks are observed at separate lengths, it is unlikely that these data can be explained by binding of bundles of fimbriae. Thus, the pulls between 2 and 10 μm likely correspond to extension of individual fimbriae.

The shapes of the force/extension curves and the constant-force regions are independent of whether 1M or 3M is on the tip, except that the 1M–FimH bond broke more often (unpublished data) due to the lower affinity of FimH to 1M relative to 3M [1]. Thus a 3M-functionalized cantilever was used for pulls probing the dynamics of fimbriae extension and contraction.

### Extension of Fimbriae Is due to the Uncoiling of Their Quaternary Structure

**Figure 3:**
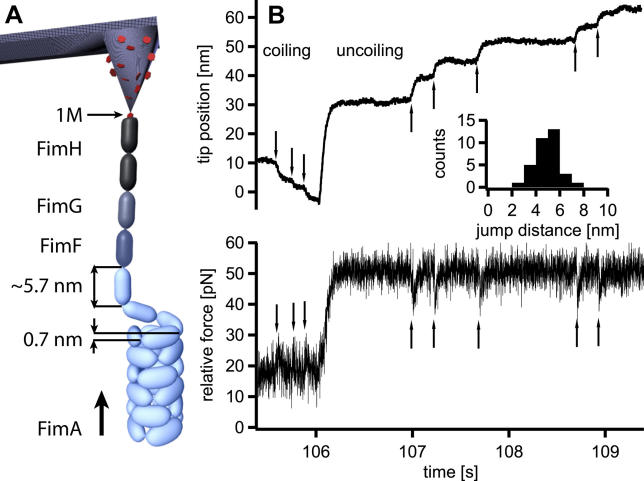
Discrete Coiling and Uncoiling Events of a Single Fimbria (A) Cartoon showing the structure of fimbriae attached to an AFM tip. The lectin domain of FimH binds specifically to mannose on the AFM tip. The fimbrial tip consists of the FimH adhesin (lectin plus pilin domain) and the FimG and FimF monomers. Below them is the 7-nm-diameter shaft (also called pilus, fimbria) made from helically coiled FimA subunits. FimA subunits contribute 0.7 nm to the length of the helical shaft [9]. By homology to the pilin domain of FimH, the length of the FimA subunit is approximately 5.7 nm. The steps measured in (B) are in good agreement with the 5-nm extension expected from (A). (B) Discrete elongation and contraction events are seen when a single fimbria is pulled at constant force just below and above the force where coiling and uncoiling rates have equal probability (*f*_bal_). The bottom trace shows that the force is temporarily lowered whenever a length increase is observed. The histogram shows the frequency of both coiling and uncoiling events combined with a peak at 5.0 ± 0.3 nm. The frequency of these single-step events increases when the applied force approaches *f*_bal,_ as expected from Equation 3. The spring constant of the cantilever used in this experiment was 6.5 pN/nm. The individual events in the histogram were obtained from four different bacteria.

Although the initial length of fimbriae cannot be measured accurately from our force/extension diagrams, electron microscopy from multiple bacterial cells of this recombinant strain revealed that the fimbrial length is typically between 0.3 and 1.5 μm (0.7 μm on average), as generally known for type I fimbriae. Two- to 10-fold stretching is consistent with the expected 7-fold extension ratio calculated from the contribution of a single subunit (0.7 nm from [9]) and the expected length of FimA from homology with the pilin domain of FimH (5.7 nm) (Figure 3A). Similar extension curves for E. coli P-fimbriae were also attributed to unraveling of the fimbrial quaternary structure [13,14]. Alternate explanations for the constant-force/extension region appear to be inconsistent with the data. Purely elastic stretching should show a linear increase in force with distance, rather than a constant force with a sudden increase in force after a particular distance. Purely entropic stretching, such as that for unraveled proteins, would require a near-zero force at short extensions. It is also unlikely that the FimA subunits are unfolding their tertiary structure, which is known to resist denaturants and even boiling. Finally, fimbrial unraveling could be seen with purified fimbriae adsorbed to a plastic surface (unpublished data), arguing against a polymerization–depolymerization mechanism attributed to the length change of type IV fimbriae [15]. The pulling of the purified fimbriae was not studied here in detail because the point of attachment to the surface changes as the fimbria peels off the surface, significantly changing the pulling angle and hence changing the actual force applied to the fimbria over the course of the pull.

To verify that the individual structural events underlying fimbrial extension are due to the uncoiling of individual FimA modules, we pulled several fimbriae near the force at which uncoiling and coiling rates balance each other (see mathematical model below) and changes in length happen slowly. To do this, we switched to a constant-force pull mode instead of the constant-velocity pull mode used above. In the constant force pulling mode, the cantilever deflection—corresponding to the desired force – is maintained at a constant level by a feedback loop that changes the position of the base of the cantilever. Figure 3B shows the position of an AFM tip over time. The trace presents discrete length changes distinctive of individual uncoiling events. The histogram in Figure 3B shows an average jump distance of 5.0 ± 0.3 nm. This is in good agreement with a structural model of the type I fimbrial rod predicting that each FimA (pilin) subunit of the rod contributes 0.7 nm to the fimbrial length in the coiled conformation [9], with the length of each FimA protein of about 5.7 nm (Figure 3A) for an expected jump distance of 5.0 nm. Although the average uncoiling jump length was found to be 0.5 nm longer than the average coiling jump, the difference was not statistically significant (*p* = 0.17). Nonetheless, such a difference might be expected, because uncoiling occurs at a force 30 pN higher than coiling, and this could stretch each FimA monomer to a greater extent. The estimated Young's modulus (a measure of elasticity) would then be of ~0.1 GPa and is consistent with that of proteins in general [16] and close to measurements in P-pili [13,14,17]. These discrete jumps confirm that the quaternary structure uncoiling of the fimbrial shaft leads to the observed extension of fimbriae as schematically illustrated in Video S1.

### Fimbriae Are Relatively Stable in Length between 25 and 60 pN

**Figure 4:**
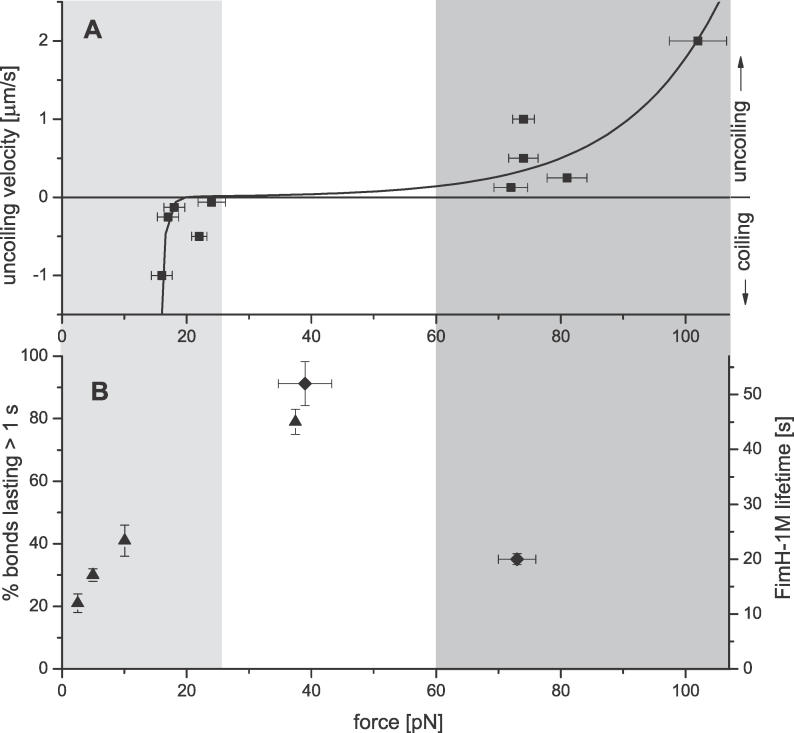
Comparison between the Uncoiling/Coiling Forces of the Fimbrial Shaft versus the Lifetime of the FimH–Mannose Bond (A) Uncoiling force of fimbriae as a function of pulling velocity (solid squares). Three regimes can be discerned: fimbriae uncoil significantly at forces greater than 60 pN (dark gray); they also coil back at significant velocities generating forces up to 25 pN (light gray); between 25 and 60 pN, fimbriae do not change significantly in length. A two-state model for uncoiling, assuming the coiled and uncoiled states are separated by a single energy barrier, was fit and is shown as a solid line. The model fits the data well and yields a height of the uncoiling and coiling energy barriers of 29*k*_b_ *T* and 4*k*_b_ *T,* respectively. The spring constant of the AFM cantilever for this experiment was of 6.7 pN/nm, and the data are taken from one bacterium (*n* > 8 data points for each point). Other bacteria probed showed the same behavior. Similar curves were also obtained by keeping the force constant and measuring the velocity. One example is shown in Figure S1. (B) Effect of force on FimH–1M bond lifetimes. The solid diamonds (right axis) show the mean lifetimes of fimbriated FimH–1M bonds measured with the AFM by holding a constant force on fimbriae and waiting for the bonds to break. The decrease of the lifetime as force increases from 40 to 60 pN shows the weakening of the bond at high forces. The solid triangles (left axis) show the fraction of FimH–1M bonds that last longer than 1 s in flow chamber experiments (*n* = 4 to 6 for each point). These experiments are described in detail in Figure 5. This fraction increases from 20% to 80% between 2.5 and 38 pN, demonstrating the catch-bond nature of FimH–1M bonds. Error bars are standard error of the mean.

A particular feature of Figure 3 is that after a change of 30 pN in force, the extension velocity changes by only approximately 20 nm/s, and this velocity is much slower than that in Figures 1B and 2, 2 μm/s. To understand what forces cause the fimbria to coil or uncoil, and how fast, we recorded the force at the constant-force region for pulling speeds from +2 μm/s (elongation) to −1 μm/s (contraction). For the contraction studies, the direction of fimbrial pulling was reversed in the middle of a pull as in Figure 2B, and the force was measured. The data in Figure 4A show a steep decrease in coiling velocity with force at low forces, a stable region between approximately 25 and 60 pN, and a gradual increase of the uncoiling velocity at higher forces. (The independent variable is set on the vertical axis for clarity with respect to the model below.) Different bacteria showed very similar curves. We also confirmed these data by maintaining a constant force and measuring the coiling or uncoiling velocities (Figure S1). Therefore, fimbriae uncoil significantly above 60 pN, coil below 25 pN, and are fairly stable in a plateau region in between.

**Figure 5:**
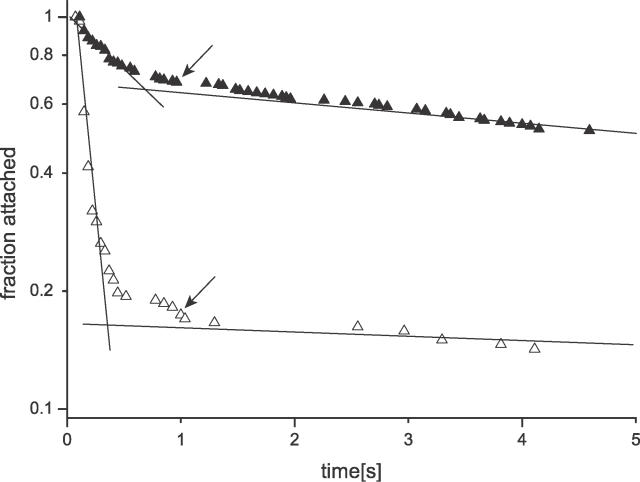
FimH–1M Bond Lifetimes in a Flow Chamber The binding of 1M beads to fimbriae-coated surfaces was monitored in flow chambers at two well-defined flow rates that create drag forces of 2.5 pN (open triangles) and 37 pN (solid triangles) on the beads. Beads moving in the field of view alternately paused (bound to the surface in a stationary manner for a period of time) and moved at the hydrodynamic velocity of the fluid flow. The lifetime of the pauses was measured and assumed to correspond to single-bond lifetimes. The lifetime of these pauses is plotted here as the fraction of all pauses that survive to the time indicated on the *x*-axis. There are two distinct decay rates under each condition, suggesting a weak and a strong conformational state, each corresponding to short-lived and long-lived binding, respectively. The fraction of pauses lasting more than 1 s (arrows) was calculated directly from raw data like these and used in Figure 4B. This fraction increased with force, consistent with the notion that FimH forms a catch bond with mannose because of a force-induced switch to a long-lived state. The lines correspond to the slopes of the exponential fits of the lifetimes in the first 500 ms or between 2 and 20 s, as described in the Materials and Methods section.

### Comparison of Force Regimes of Fimbrial Dynamics and FimH–Mannose Bond Strength

Because fimbriae are the supporting structures through which force is transmitted to the adhesin FimH, we next addressed how extension or contraction of the fimbrial shaft may affect the fimbriated FimH–mannose bond lifetime and, thus, bacterial surface binding. Our previous work has provided evidence that FimH forms a biphasic catch bond with mannose [4], defined as a bond that switches from low to high binding under force. If averaged over many events, it manifests itself in a biphasic adhesion curve for which the averaged lifetime first increases with increasing force up to a maximum and then decreases. We therefore ask how coiling and uncoiling forces relate to the forces at which catch bonds present either weak or strong adhesive properties.

To measure how long the fimbriated FimH–1M bonds last under medium (presumably, optimal) and high levels of force, we used the closed-loop feedback to maintain a constant force on the FimH–1M bond, and we measured how long the bond lasted. Only the events for which a single fimbria was attached to the AFM cantilever were analyzed. The mean bond lifetime was 52 ± 4 s at 40 pN and 20 ± 1 s at 70 pN (Figure 4B, diamonds), showing that the bond lifetime shortens above 40 pN. Comparison to Figure 4A shows that when the lifetime is large at medium forces (40 pN), there is not much uncoiling. However, uncoiling is significant at the higher forces where the lifetime of the bond is reduced (Figure 4A, dark gray shaded region).

There are two possible ways to measure the lifetimes of the FimH–mannose bonds at low forces: (i) by directly pulling on bacteria at a low constant force, or (ii) by first extending fimbriae and then applying the low constant force. However, both of these approaches require the AFM tip to be near the bacterium for long periods of time (in the second case because fimbriae recoil and return the tip close to the bacterium) during which other fimbriae can attach (unpublished data). Thus, it is not straightforward to measure lifetimes of fimbriated FimH–mannose bonds at low forces. We used an alternative approach and measured the time that 1M-coated polystyrene beads paused on the surface of flow chambers that were coated with fimbriae sheared off of bacteria in an assay similar to that in [3]. This flow-cell–based assay has better low-force resolution than the AFM and has given comparable results to the AFM when used previously with P-selectin catch bonds [18]. Because the radius *(r)* of the beads is known, the drag force can be calculated given the shear stress τ using Goldman's formula (F = 1.7 × 6 × πτ*r* ^2^ [19]).

To find the low-force regime at which the lifetime of the fimbriated FimH–1M bond increases (a characteristic feature of catch bonds), we tracked the trajectories of the beads above to identify when and for how long they paused. The fraction of pauses surviving as a function of time (Figure 5) showed a pronounced double exponential decay, with many beads beginning to move again in under 80 ms and the remainder in well over 4 s. That is, there was a >50-fold difference in the characteristic lifetimes of the short and long pauses. A change in fimbriae concentration or 1M concentration had no measurable effect on the fraction of short and long pauses or on their durations (unpublished data), strongly suggesting that both short and long pauses reflect single bonds. This supports the structural model, inspired by the structural changes of FimH under force in steered molecular dynamic simulations [1]. This model assumes that the FimH–1M bond can be in two states: a weak, short-lived state and a strong, long-lived binding state. When the fraction of long-lived bonds (lasting >1 s) is plotted as a function of force, this fraction increases dramatically between 2.5 and 38 pN (Figure 4B, triangles) from 21 ± 3% to 79 ± 4%. It is this force-induced switch from the short-lived to the long-lived state that indicates that FimH–1M forms a catch bond. This behavior was also seen in our earlier report [4], but the use of beads here allows for better quantification of drag forces than with the nonspherical and fimbriated bacteria. A comparison between Figure 4A and 4B shows that significant uncoiling (dark gray area) occurs only at forces high enough to switch most FimH–1M bonds into the strong state. Similarly, significant recoiling (light gray) would occur if the force drops too low to cause this switch. This means that the FimH–1M bonds should usually switch into the strong state in the AFM fimbrial pulling experiments reported here, explaining why most interactions measured in these AFM fimbrial pulls are long lived but a small number are very short lived.

Combining the observations from the AFM and the flow chambers, we conclude that average bond lifetimes are longest in the region in which fimbrial length is stable (Figure 4, region not shaded). At lower coiling forces, the force is not high enough to switch most bonds to a long-lived state before they detach, whereas at higher uncoiling forces, the force shortens the lifetime of even the long-lived state.

### Quantifying the Dynamics of Fimbrial Uncoiling

To quantify the parameters of the coiling and uncoiling processes, we developed a model for fimbrial dynamics at different forces. It explains the data well, including the discrete step size and rate at near-equilibrium forces using parameters consistent with the structural knowledge about fimbriae. The mathematical model we present assumes that coiling and uncoiling occur only at the tip end of the helix. Uncoiling in the coiled section of the helix requires the simultaneous break of the four involved subunits, requiring four times the activation energy for a single uncoiling event, and is significantly less likely (Equation 1). Thus uncoiling is most probably nucleated at the tip, and indeed a partially uncoiled tip is seen in electron microscopy studies [9]. Similarly, the energy required to coil four sequential subunits instead of only one against a force in order to make a stable turn makes coiling in parallel relatively unlikely.

On the basis of the discrete jumps in Figure 3B, we assumed that each subunit can be in two possible states: a coiled state, in which it is bound to the FimA subunits in the next turn of the helix, or an uncoiled state, in which it has contact with the neighboring subunits in the linear polymer only via the swapped β strands (Figure 3A). If we assume that there is a single transition state at which the FimA–FimA contact is equally likely to break or form, then uncoiling of a FimA subunit is expected to happen stochastically at a rate *k*_U_(*f*). This rate depends on the energy barrier height between the coiled and transition states Δ*U*_U_ as well as the difference in length between these two states Δ*x*_U_ and the applied force *f,* as described by the Bell model [20] for a noncovalent bond:


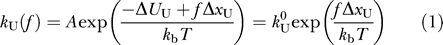


Here, *A* is the Arhenius frequency factor, *k*_b_ *T* is the thermal energy, and 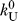 is the uncoiling rate in the absence of force. There will be an analogous rate equation for *k*_C_(*f*) for coiling depending on the energy Δ*U*_C_ and distance Δ*x*_C_ differences between the uncoiled and transition states. From the single transition state assumption, we get that Δ*x*_C_ = Δ*x*_U_ − Δ*L* and Δ*U*_C_ = Δ*U*_U_ − Δ*U*, where Δ*U* is the energy cost to uncoil a monomer and Δ*L* is the extension due to one subunit uncoiling.

With this model, we can relate the average extension velocity *V* to the applied force


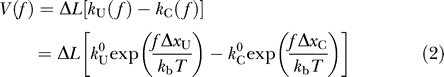


This equation should hold whether the AFM tip pulls the fimbrial tip at a constant velocity while recording the force (Figures 1, 2, and 4A), or the tip maintains a constant force while the position is recorded (Figures 3B and S1). A consequence of Equation 2 is that there is a force, *f*_bal_, at which the average velocity is zero and the uncoiling and coiling rates balance each other [*k*_bal_ = *k*_U_(*f*_bal_) = *k*_C_(*f*_bal_)]. We can then rewrite Equation 2 as


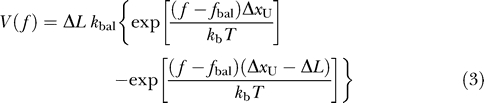


We fit this model to the data in Figure 4A using Δ*L* = 5.0 nm and *k*_bal_ = 2.2 s^−1^ as calculated from data in Figure 3B. The least-squares fit (Figure 4A, solid line) estimated *f*_bal_ = 20 ± 2 pN and Δ*x*_U_ = 0.26 ± 0.01 nm. This corresponds to an uncoiling energy barrier of 29*k*_b_ *T* and a coiling barrier close to 4*k*_b_ *T*. The model also predicts a range of forces (25 to 60 pN) for which uncoiling is slow, consistent with the force range at which slow uncoiling was observed in Figure 4A. Thus, the pattern of coiling, stable plateau, and uncoiling shown in Figure 4A is predicted by a biophysical model of quaternary uncoiling.

## Discussion

When force is applied via the adhesin-bound ligand to the terminal end of type I fimbriae, the fimbriae extend and contract reversibly. When type I fimbriae are pulled at constant velocity, the force/extension curves show a constant-force region where the fimbriae extend severalfold, and this force increases with the pulling velocity. The data show that fimbriae are fairly stable in length (contracting or extending at a velocity of less than 0.1 μm/s) over a range of forces between 60 and 25 pN, but extend or contract rapidly outside this range. When the fimbriae are pulled at constant force within this intermediate region, discrete elongation and contraction events are observed whose extension is remarkably close to the geometric length increase expected for single FimA subunits uncoiling from the helical shaft and orienting themselves along the force vector. Together, the 5-nm individual elongation events and the dynamic behavior confirm that the mechanism of fimbrial contraction and elongation is reversible and originates from the coiling and uncoiling of the helical shaft. A two-state model for the dynamics of uncoiling was derived and accounts well for our experimental findings. It yields an uncoiling energy barrier of 29*k*_b_ *T,* and a force of 22 pN at which coiling and uncoiling events occur at equal probability, i.e., the maximum force fimbriae can generate when coiling. We showed with AFM and flow cell measurements that the lifetime of individual fimbriated FimH–1M complexes is long when pulled at the intermediate constant forces at which fimbriae change little in length but is shorter at low and high forces that cause significant coiling and uncoiling.

An explanation for this relationship between the kinetics of type I fimbriae and fimbriated FimH–1M bond lifetimes is that they have codeveloped well-matched kinetic properties. This potentially explains the observation that changing the sequence of FimA in the fimbrial shaft affects binding [21]. Coiling and uncoiling could maximize adhesion under flow in a number of ways. Although the kinetic parameters were derived here by applying constant forces, the flow rates often change under physiological conditions. Uncoiling or coiling could then modulate the force experienced by the adhesin–ligand complex as well as the loading rates, which affect the bond lifetimes and breaking forces [20,22]. First, as we show here, fimbriae can keep the force constant on the FimH–mannose bonds during short surges in shear stress while they uncoil, absorbing the energy imparted by drag force and effectively acting as a damper, because each uncoiling event absorbs 25*k*_b_ *T* (~40 kcal/mol). When shear diminishes, coiling can generate forces in the order of 20 pN, which can hoist the bacterium back to its initial anchoring point. It is also possible that a bacterium can drag along the surface during uncoiling and has a chance to form other bonds or share the load among fimbriae, which could be critical when the receptor density is low or the shear is high. Then, when the angle between the surface and the tether is nonzero, the cell acts as a lever arm that increases the bond force [23,24]. Uncoiling reduces the angle and thus reduces the force applied to fimbriae by fluid drag forces.

Taken together, these results describe why it is advantageous for the fimbriae to start uncoiling after the FimH–1M lifetime peaks (as shown in this study), not before. Thus, the mechanical properties of the helical quaternary structure of fimbriae appear to be designed to maximize the fraction of FimH–mannose bonds in the long-lived state, thereby maximizing bacterial adhesion to surfaces in a wide range of flow conditions. The P-fimbriae that were shown previously to unravel under force [13,14,17] were also shown to display shear-enhanced behavior [25] when bound through their terminal adhesive subunit ligand. Although the structural mechanism of shear enhancement for P-fimbriae has not been determined, there is extensive evidence that shear enhancement of FimH–1M adhesion is due in large part to catch-bond properties of the FimH–1M complex.

In contrast to type IV pili [26], type I fimbrial extrusion happens in the absence of ATP [27], when linearly arranged FimA units in the FimD pore [28] coil on the outer membrane. The energy gained upon addition of a FimA unit to the quaternary helix according to the model in the Results section is 25*k*_b_ *T,* close to that gained upon hydrolysis of an ATP molecule. This energy gain from coiling may help drive extrusion of type I fimbriae from the periplasm in what is known as the chaperone/usher pathway [27].

A striking parallel emerges when we consider another known catch bond system, P-selectin [18]. In this system, membrane tether formation induced by force [23,24,29] might play a similar role to fimbrial uncoiling, which suggests that the attachment of catch bond–forming receptor–ligand complexes on extensible tethers might be a common motif that enhances the ability of cells to bind to surfaces under shear conditions. The structures of many proteins codevelop to maximize their biochemical interactions, but this work suggests that the mechanical properties of FimA and the FimH–mannose complex have codeveloped to optimize bacterial adhesion under flow. This also stresses the notion that the response of mechanically active molecules such as FimH or selectins can be modulated by their supporting structures at multiple levels [30–32]. Our results demonstrate that the fimbrial rod has more functions than simply acting as an inert structure that only protrudes the tip-associated adhesive subunit, FimH, away from the capsule [5] or help overcome repulsive forces from surfaces [6] as proposed previously.

## Materials and Methods

### Bacterial strains.

Recombinant strains used here were constructed using a *fim*-null E. coli K-12 derivative AAEC191A, as described in [33]. AAEC191A was transformed with the recombinant plasmid pPKL114 that contains the entire *fim* gene cluster from the E. coli K-12 strain, PC31, but with a translational stop linker inserted into the *fimH* gene (provided by P. Klemm, Danish Technical University, Copenhagen). These were cotransformed with a pGB2–24–based plasmid containing a *fimH* allele identical to that in E. coli K-12 (FimH-j-96), under the *bla* promoter. Recombinant strains created using these plasmids express fully functional type I fimbriae that are morphologically identical to the ones expressed by wild-type bacteria.

### Surface functionalization.

Plain glass coverslips or glass slides were functionalized with 50 μl of 100 μg/ml ribonuclease B (RB) from bovine pancreas obtained from Sigma (R7884) (St. Louis, Missouri, United States) in a 0.02 M bicarbonate buffer (pH 9) incubated for 75 min at 37 °C. RB is a model 3M receptor that binds strongly to FimH. The slides were then rinsed three times in phosphate buffered saline (PBS) solution and the nonfunctionalized region was dried. Another 50 μl of E. coli at a concentration of ~10^9^ bacteria/ml in PBS was deposited on the slide and incubated at 37 °C for 1 h. Unbound bacteria were washed by rinsing again in PBS.

### AFM measurements.

Tip functionalization: Biolever type B cantilevers from Olympus (Tokyo, Japan) (nominal spring constant of 6 pN/nm) were incubated in a 100 μg/ml solution of RB or monomannosylated bovine serum albumin (BSA) (gift of Y. C. Lee, Johns Hopkins University, Baltimore, Maryland, United States, used as a model 1M receptor) for 1 h and immediately used for experiments after washing in PBS buffer. The spring constant of the cantilevers was calibrated using the thermal method. The position of the cantilever base (same as the piezo position) was recorded by means of an integrated linear voltage displacement transformer of sub-nm accuracy and a nonlinearity less than 0.2% (Average deviation/full travel) at full scan. The factory calibration was checked with a 180-nm grid and found to be within specifications. Constant velocity pulls were done in the closed-loop mode with an Asylum Research MFP 3D AFM (Santa Barbara, California, United States) by making the piezo velocity constant. The actual velocity of the tip is the velocity of the piezo minus the velocity at which the cantilever deflects from its equilibrium position. For the constant-force region, the change in deflection was minimal (<2 nm/μm), so its velocity was negligible (<1%) relative to the total velocity. In the regions before and after the constant-force region, the contribution of the cantilever deflection reduced the velocity of the tip up to 10%, but because no data were used for analysis in these sections, no corrections were made.

The pull in Figure 2A was done by extending fimbriae at 2 μm/s until all fimbriae detached. The pull in Figure 2B was obtained by first pulling for 2 μm. Then the cantilever turned back, approaching the bacterium at a set coiling velocity for 1 μm before it turned back again and retracted at a set uncoiling velocity. The force measured during these coiling and uncoiling constant-force regions was then plotted in Figure 4A for different velocities. In order to measure lifetimes, we pulled on fimbriae at a set velocity until only a single tether held. Then we used the closed-loop feedback to maintain a constant deflection on the cantilever, corresponding to a force of either 40 or 70 pN, and measured how long the bond lasted (Figure 4B). The resulting histogram of lifetimes was integrated to obtain the survival function of the bonds, and then was fit to a single exponential decay with a constant term to account for nonspecific events. The characteristic time of these fits is reported as the lifetime of the bonds. These data were obtained by probing four different bacteria multiple times at both 40 and 70 pN with the same cantilever of spring constant 7.9 pN/nm. All experiments were done at room temperature.

### Flow chamber experiments.

Polystyrene microspheres with diameters of 3 μm and coated with 200 μg/ml 1M-BSA were continually injected into a parallel plate flow chamber coated with 0.1–0.01 μg/ml K12 fimbriae as described previously [3]. After 2 min of injection at a shear stress of 0.3 pN/μm^2^, the flow chamber was washed at 1 pN/μm^2^ to remove free-floating beads. Then the flow was turned off for 10 min to unbind all beads. PBS with 0.2% BSA by weight was then pumped through the chamber with the desired flow rate. The movement of the beads was recorded at each shear stress with a charge-coupled device camera and MetaMorph (Molecular Devices, Sunnyvale, California, United States) video acquisition software at 37 ms per frame for 20 s. The beads in the videos were tracked using SVision's Image Based Decision technology (SVLife, Seattle, Washington, United States) as described in [4]. The minimum pause time measurable was 74 ms (two frames) with increments of 37 ms, because pauses could be conclusively detected only if the bacteria remained in the same position for two consecutive frames. In this manner, all the pause lifetimes were measured to the nearest 37 ms. From this, a histogram of pause lifetimes could be obtained, which was then integrated to obtain the survival plot in Figure 5 as previously done with bacteria [4]. The lifetimes of the pauses were fit in two steps. From 0 to 500 ms, a single exponential fit with a constant term provided the lifetime of the short state. This lifetime was close to 60 ms for most datasets, with the exception of the high-shear dataset (38 pN), where the low proportion of short lifetime events affected the measurement. From 2 to 20 s, a single exponential fit resulted in lifetimes that varied between 4 and 80 s in the different datasets. Here the quality of the fit was strongly affected by the number of beads remaining at the 2-s time point, which in turn depended on the proportion of long-lived events and the initial number of beads. Fimbriae preparation, tissue plate coating, and bead coating were performed according to protocols described previously [3]. Briefly, fimbriae were sheared off bacteria by a homogenizer followed by differential centrifugation and magnesium chloride precipitation; 35-mm Corning (Corning, New York, United States) tissue culture dishes were incubated with purified fimbriae at 0.1–0.01 μg/ml in 0.02 M bicarbonate buffer at 37 °C for 1 h and then washed three times in PBS with 0.2% BSA (PBS-BSA) to prevent nonspecific adhesion by the beads to the dish; 3-μm polystyrene microspheres were prepared by rotating a solution of 50 μl of 2.6% beads mixed with 150 μl of 100 μg/ml 1M-BSA in 0.02 M bicarbonate buffer for 1 h at 37 °C. The beads were then spun twice and resuspended in fresh PBS-BSA. They were finally diluted down to 0.1% and injected in the chamber.

## Supporting Information

Figure S1 Fimbria Uncoiling Dynamics at Constant Forces Force versus uncoiling velocity curve for a single bacterium obtained using the constant-force mode of the AFM. These data confirm the data in Figure 4A with a different method. This curve appears shifted by 5–10 pN to lower forces with respect to Figure 4A, possibly because these data were taken on a single pull, whereas Figure 4 reflects the average of at least eight different measurements. Also, the zero level of the force in the constant-force mode is less accurate due to a larger effect of drift in these longer duration pulls.
(249 KB JPG)

Video S1 Cartoon Movie Illustrating the Uncoiling of a Fimbria by an AFM Tip A mannose-covered AFM tip attaches to the terminal FimH subunit of a fimbria. The cantilever bends as the pulling force on the fimbria increases. When the force is sufficient, individual FimA subunits uncoil in discrete steps from the fimbrial shaft, consistent with the distance jumps in Figure 3B.
(3.6 KB AVI)

## Abbreviations

1M: monomannose
3M: trimannose
AFM: atomic force microscope
BSA: bovine serum albumin
PBS: phosphate buffered saline

## References

[pbio-0040298-b001] Thomas WE, Trintchina E, Forero M, Vogel V, Sokurenko EV (2002). Bacterial adhesion to target cells enhanced by shear force. Cell.

[pbio-0040298-b002] Thomas WE, Nilsson LM, Forero M, Sokurenko EV, Vogel V (2004). Shear-dependent 'stick-and-roll' adhesion of type 1 fimbriated Escherichia coli. Mol Microbiol.

[pbio-0040298-b003] Forero M, Thomas WE, Bland C, Nilsson LM, Sokurenko EV (2004). A catch-bond based nanoadhesive sensitive to shear stress. Nano Lett.

[pbio-0040298-b004] Thomas W, Forero M, Yakovenko O, Nilsson L, Vicini P (2006). Catch-bond model derived from allostery explains force-activated bacterial adhesion. Biophys J.

[pbio-0040298-b005] Schembri MA, Dalsgaard D, Klemm P (2004). Capsule shields the function of short bacterial adhesins. J Bacteriol.

[pbio-0040298-b006] Marshall KC (1986). Adsorption and adhesion processes in microbial growth at interfaces. Adv Colloid Interface Sci.

[pbio-0040298-b007] Busscher HJ, Weerkamp AH (1987). Specific and non-specific interactions in bacterial adhesion to solid substrata. FEMS Microbiol Lett.

[pbio-0040298-b008] Klemm P (1984). The fimA gene encoding the type-1 fimbrial subunit of *Escherichia coli.* Nucleotide sequence and primary structure of the protein. Eur J Biochem.

[pbio-0040298-b009] Hahn E, Wild P, Hermanns U, Sebbel P, Glockshuber R (2002). Exploring the 3D molecular architecture of Escherichia coli type 1 pili. J Mol Biol.

[pbio-0040298-b010] Sauer FG, Futterer K, Pinkner JS, Dodson KW, Hultgren SJ (1999). Structural basis of chaperone function and pilus biogenesis. Science.

[pbio-0040298-b011] Choudhury D, Thompson A, Stojanoff V, Langermann S, Pinkner J (1999). X-ray structure of the FimC-FimH chaperone-adhesin complex from uropathogenic Escherichia coli. Science.

[pbio-0040298-b012] Abraham SN, Land M, Ponniah S, Endres R, Hasty DL (1992). Glycerol-induced unraveling of the tight helical conformation of Escherichia coli type 1 fimbriae. J Bacteriol.

[pbio-0040298-b013] Jass J, Schedin S, Fallman E, Ohlsson J, Nilsson UJ (2004). Physical properties of Escherichia coli P pili measured by optical tweezers. Biophys J.

[pbio-0040298-b014] Fallman E, Schedin S, Jass J, Uhlin BE, Axner O (2005). The unfolding of the P pili quaternary structure by stretching is reversible, not plastic. EMBO Rep.

[pbio-0040298-b015] Maier B, Koomey M, Sheetz MP (2004). A force-dependent switch reverses type IV pilus retraction. Proc Natl Acad Sci U S A.

[pbio-0040298-b016] Howard J (2001). Mechanics of motor proteins and the cytoskeleton.

[pbio-0040298-b017] Andersson M, Fallman E, Uhlin BE, Axner O (2006). A sticky chain model of the elongation and unfolding of Escherichia coli P pili under stress. Biophys J.

[pbio-0040298-b018] Marshall BT, Long M, Piper JW, Yago T, McEver RP (2003). Direct observation of catch bonds involving cell-adhesion molecules. Nature.

[pbio-0040298-b019] Goldman AJ, Cox RG, Brenner H (1967). Slow viscous motion of a sphere parallel to a plane wall—II Couette flow. Chem Eng Sci.

[pbio-0040298-b020] Bell GI (1978). Models for the specific adhesion of cells to cells. Science.

[pbio-0040298-b021] Duncan MJ, Mann EL, Cohen MS, Ofek I, Sharon N (2005). The distinct binding specificities exhibited by enterobacterial type 1 fimbriae are determined by their fimbrial shafts. J Biol Chem.

[pbio-0040298-b022] Evans E, Ritchie K (1997). Dynamic strength of molecular adhesion bonds. Biophys J.

[pbio-0040298-b023] Smith MJ, Berg EL, Lawrence MB (1999). A direct comparison of selectin-mediated transient, adhesive events using high temporal resolution. Biophys J.

[pbio-0040298-b024] Schmidtke DW, Diamond SL (2000). Direct observation of membrane tethers formed during neutrophil attachment to platelets or P-selectin under physiological flow. J Cell Biol.

[pbio-0040298-b025] Nilsson LM, Thomas WE, Trintchina E, Vogel V, Sokurenko EV (2006). Catch bond-mediated adhesion without a shear threshold: Trimannose versus monomannose interactions with the FimH adhesin of Escherichia coli. J Biol Chem.

[pbio-0040298-b026] Soto GE, Hultgren SJ (1999). Bacterial adhesins: Common themes and variations in architecture and assembly. J Bacteriol.

[pbio-0040298-b027] Thanassi DG, Stathopoulos C, Karkal A, Li H (2005). Protein secretion in the absence of ATP: The autotransporter, two-partner secretion and chaperone/usher pathways of gram-negative bacteria (review). Mol Membr Biol.

[pbio-0040298-b028] Saulino ET, Bullitt E, Hultgren SJ (2000). Snapshots of usher-mediated protein secretion and ordered pilus assembly. Proc Natl Acad Sci U S A.

[pbio-0040298-b029] Girdhar G, Shao JY (2004). Membrane tether extraction from human umbilical vein endothelial cells and its implication in leukocyte rolling. Biophys J.

[pbio-0040298-b030] Heinrich V, Leung A, Evans E (2005). Nano- to microscale dynamics of P-selectin detachment from leukocyte interfaces. II. Tether flow terminated by P-selectin dissociation from PSGL-1. Biophys J.

[pbio-0040298-b031] Evans E, Heinrich V, Leung A, Kinoshita K (2005). Nano- to microscale dynamics of P-selectin detachment from leukocyte interfaces. I. Membrane separation from the cytoskeleton. Biophys J.

[pbio-0040298-b032] King MR, Heinrich V, Evans E, Hammer DA (2005). Nano-to-micro scale dynamics of P-selectin detachment from leukocyte interfaces. III. Numerical simulation of tethering under flow. Biophys J.

[pbio-0040298-b033] Sokurenko EV, Courtney HS, Maslow J, Siitonen A, Hasty DL (1995). Quantitative differences in adhesiveness of type 1 fimbriated Escherichia coli due to structural differences in fimH genes. J Bacteriol.

